# Comparison of geographical and individual deprivation index to assess the risk of Sars-CoV-2 infection and disease severity: a retrospective cohort study

**DOI:** 10.1186/s12942-024-00367-6

**Published:** 2024-04-04

**Authors:** Sara Mazzilli, Gianluca Paparatto, Antonio Chieti, Anna Maria Nannavecchia, Lucia Bisceglia, Pier Luigi Lopalco, Domenico Martinelli, Lara Tavoschi

**Affiliations:** 1 Scuola Normale Superiore, Pisa, Italy; 2 Department of Translational Research and New Technologies in Medicine and Surgery, University of Pisa, Pisa, Italy; 3 Health Science Interdisciplinary Research Centre, Sant’Anna School of Advanced Studies, Pisa, Italy; 4 Strategic Regional Health and Social Agency of Puglia (AReSS Puglia), Bari, Italy; 5 Department of Biological and Environmental Sciences and Technology, University of Salento, Lecce, Italy; 6 Hygiene Unit, Policlinico Foggia Hospital, Department of Medical and Surgical Sciences, University of Foggia, Foggia, Italy

**Keywords:** Socioeconomic disadvantage, Deprivation index, Sars-CoV-2, COVID-19, Apulia, Italy

## Abstract

**Background:**

It has been shown that COVID-19 affects people at socioeconomic disadvantage more strongly. Previous studies investigating the association between geographical deprivation and COVID-19 outcomes in Italy reported no differences in case-hospitalisation and case-fatality. The objective of this research was to compare the usefulness of the geographic and individual deprivation index (DI) in assessing the associations between individuals' deprivation and risk of Sars-CoV-2 infection and disease severity in the Apulia region from February to December 2020.

**Methods:**

This was a retrospective cohort study. Participants included individuals tested for SARS-CoV-2 infection during the study period. The individual DI was calculated employing polychoric principal component analysis on four census variables. Multilevel logistic models were used to test associations between COVID-19 outcomes and individual DI, geographical DI, and their interaction.

**Results:**

In the study period, 139,807 individuals were tested for COVID-19 and 56,475 (43.5%) tested positive. Among those positive, 7902 (14.0%) have been hospitalised and 2215 (4.2%) died. During the first epidemic wave, according the analysis done with the individual DI, there was a significant inversely proportional trend between the DI and the risk of testing positive. No associations were found between COVID-19 outcomes and geographic DI. During the second wave, associations were found between COVID-19 outcomes and individual DI. No associations were found between the geographic DI and the risk of hospitalisation and death. During both waves, there were no association between COVID-19 outcomes and the interaction between individual and geographical DI.

**Conclusions:**

Evidence from this study shows that COVID-19 pandemic has been experienced unequally with a greater burden among the most disadvantaged communities. The results of this study remind us to be cautious about using geographical DI as a proxy of individual social disadvantage because may lead to inaccurate assessments. The geographical DI is often used due to a lack of individual data. However, on the determinants of health and health inequalities, monitoring has to have a central focus. Health inequalities monitoring provides evidence on who is being left behind and informs equity-oriented policies, programmes and practices. Future research and data collection should focus on improving surveillance systems by integrating individual measures of inequalities into national health information systems.

**Supplementary Information:**

The online version contains supplementary material available at 10.1186/s12942-024-00367-6.

## Introduction

The newly emerged virus SARS-CoV-2 was initially reported in China in December 2019 [1]. On February 20, 2020, the first major COVID-19 outbreak in Europe was detected in the Lombardy region, Italy [2]. On March 11, 2020, WHO declared the SARS-CoV-2 outbreak a pandemic [3]. As of August 30, 2023, the WHO reports a total of 770,085,713 confirmed cases and 6,956,173 confirmed deaths worldwide [4]; while the Italian NHS reported 26,175,146 confirmed cases, and 190,644 deaths [5]. During the first pandemic year, the temporal course of the epidemic in Italy was characterized by 3 distinct phases: the first epidemic wave from March to June 2020, followed by a summer period with a relatively low incidence, and a second wave that started in September and peaked in November 2020 [4].

Evidence shows that males, aged over 65 and smoking patients might face a greater risk of developing more critical or lethal conditions if infected with SARS-CoV2. Comorbidities, such as hypertension, diabetes, cardiovascular disease or respiratory diseases, could also greatly affect the prognosis of COVID-19 patients [6]. A study carried out in a large community cohort has also shown associations between adverse lifestyles and a higher risk of COVID-19 [7]. However, it is now well known that lifestyle plays a mediating role in the relationship between socioeconomic position (SEP) and health [8]. Literature reports that the wide inequalities seen in infection, hospitalization and mortality rates between population groups were mostly driven by social factors overlaid on biological risks [9, 10]. Multiple mechanisms explain the increased impact of COVID-19 among people with a higher level of socio-economic disadvantage, but in summary, unfavourable social determinant of health (SDH) and associated higher rates of chronic disease [11] increased their risk of poor outcomes from COVID-19 and poorer access to health services for treatment and vaccination [12]. While existing predisposing susceptibility to COVID-19 is a product of pre-existing SDH, there is growing evidence that the ability of disadvantaged groups to adhere to public health and social measures that reduce viral transmission and to deal with the aftermath of the pandemic, is also negatively affected by unfavourable SDH [13]. Difficult living and working conditions make adherence to preventive measures more difficult for disadvantaged populations, thus increasing their exposure to the risk of infection [14]. For instance, most deprived individuals usually carry out manual labour professions or work in the informal sector and therefore, resulting in limited opportunities for working from home. Health and socioeconomic inequality mutually influence each other by triggering and feeding vicious circles [12]. The economic fallouts from the pandemic are hitting disadvantaged population groups harder. The pandemic itself has accentuated these already existing social and health inequalities, widening the gap among individuals with different SEP. This is a crucial point for the present but also for the future. Assessment and mitigation of SDH cannot be neglected in a pandemic response and prevention programme.

The available Italian deprivation index (DI) is a multidimensional measure of the disadvantage in the ownership of both social and material resources among residents in each census sections, which are comparable to neighbourhoods as described elsewhere [15, 16]. Mateo-Urdiales et al. investigated the association between deprivation and COVID-19 outcomes in Italy during pre-lockdown, lockdown and post-lockdown periods using the Italian DI as a contextual measure of deprivation. No differences in case-hospitalisation and case-fatality according to deprivation were observed [17]. Similarly, in a study on the city of Barcelona, the increase in hospitalisation and mortality rates was not significant among people living in areas characterised by higher geographical DI [18]. However, the use of geographical DI measures as a proxy for the level of individual social disadvantage is subject to potential ecological bias that can arise from attributing a collective measure to an individual.

The overall objective of this research was to compare the geographic and individual DI in assessing the associations between individuals' SEP and risk of Sars-CoV-2 infection and disease severity in the Apulia region from February to December 2020.

## Methods

### Study setting

The Italian’Servizio Sanitario Nazionale’ (SSN) was introduced in 1978 to ensure that healthcare is accessible to all Italian citizens without socio-economic barriers, according to a principle of horizontal equity [19]. The system is organized into three levels: national, regional, and local. The national level is responsible for establishing the general objectives and fundamental principles of the NHS. The nineteen regions and two autonomous provinces (R&AP) are then responsible for organizing and delivering health care [20]. In this scenario, through ministerial decrees, the Ministry of Health has taken the lead in the fight against the COVID-19 epidemic. Then, the R&AP were in charge of organizing and implementing the monitoring and prevention strategy at the local level based on national guidelines. This study is carried out in the Apulia region (Dimension: 19.540 km2), which is a region in southern Italy as shown in red in Additional file 1: Figure S1. As of December 31, 2019, the population of the Apulia region was 3,953,305. The region is administratively divided into six provinces, each with its own LHA. In 2019, 51.4% of the population in Apulia were females, and 3.4% were foreigners. The average age of the population was 45.4 years, with individuals aged over 65 accounting for 23.1% of the entire population. Additionally, 40.4% of the population had at least one chronic disease, with the most common conditions being hypertension (18.8%), arthrosis and arthritis (17.0%), allergic diseases (11.8%), and osteoporosis (9.4%) [21]. In Apulia region, the hospital network comprises 33 public and 26 private institutions, providing a total of 11,565 beds for ordinary inpatient care, equating to 2.93 beds per 1000 people.

### Pandemic stages and preventive measures

In Italy, after the detection of the first locally acquired Covid-19 case in Lombardy on February 20, 2020, the number of cases increased greatly in the following weeks, although unevenly among regions, forcing the government to adopt unprecedented restrictive measures. In particular, during the 1st year of the pandemic, the following phases can be defined: 1. first comprehensive national lockdown from 9 March to 3 May (closure of schools and most workplaces, and the implementation of quarantines, border closings, and restriction on public gatherings) 2., 22] gradual reopening phase from 4 May to 14 June (restrictions were gradually rolled back) 3. , 23] Few restrictions from 15 June to 7 October (Covid-19 incidence remained low) 4. , 24] new restrictions from 8 October to 5 November [25], and 5. lockdowns on a regional basis from 6 November until the end of 2020 (closure of regional borders) [26]. During the first epidemic wave, the number of diagnostic tests (Polymerase Chain reaction—PCR) available was limited, while availability increased considerably during the second wave [27].

### Study design and data sources

**Fig. 1:**
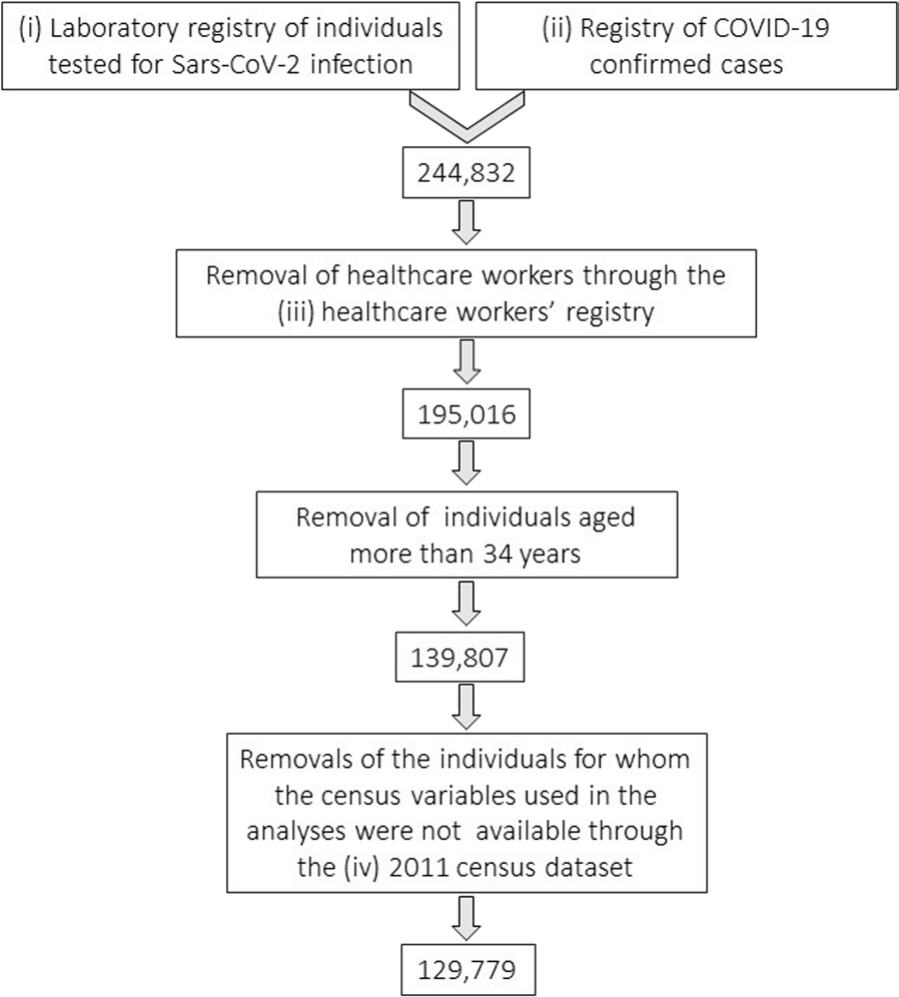
Data linkage to obtain the study cohort

This is a retrospective cohort study conducted by analysing and merging the following electronic health records from the Apulia regional health-care information systems: (i) laboratory registry of individuals tested for Sars-CoV-2 infection; (ii) registry of COVID-19 confirmed cases; (iii) healthcare workers’ registry; (iv) 2011 census dataset. Figure 1. explains how the final dataset was obtained. In the study, a COVID-19 confirmed case was defined as an individual who resulted positive to a PCR test. The study period goes from 20 February to 31 December 2020. The cohort in this study includes all the individuals tested for SARS-CoV-2 infection during the study period in the Apulia region, in which a total of 3.95 million inhabitants were registered as of December 31, 2019 [28]. Since the census data date back to 2011, we decided to exclude persons under 35 years of age, for whom part of the census data (i.e. education) may have changed. Individuals tested from 20 February 2020 to 31 May 2020 were included in the first epidemic wave, while those tested from 16 September 2020 to 31 December 2020 were included in the second epidemic wave [4].

The laboratory registry tracked: identification code; age; sex; province of residence; date of the test; PCR test result. The COVID-19 confirmed cases registry reported the following information: identification code, COVID-19 related hospitalization; admission to the intensive care unit (ICU) due to COVID-19 and death due to COVID-19. From the census variables available, the following were selected to perform this study: identification code; education; the number of family members; citizenship; family type; and employment status.

Charlson comorbidities index, extensively described previously [29], and the geographical DI [15, 16] were added to the characteristics of individuals in the final dataset. The Italian geographical DI is the sum of the z score of five simple indicators: x_1_: % of population among 15 and 60 years old with education equal to or less than elementary school; x_2_: % of the active population unemployed or seeking their first job; x_3_: % of housing occupied for rent; x_4_: % of single-parent families (and consisting of a single-family unit) with children under 18 years old; x_5_: population density (occupants per 100 m2).

Through the use of the healthcare workers’ registries, healthcare workers were removed from the dataset as their risk of acquisition of infection was strongly related to their professional exposure to the virus.

The merging of the data information system has been carried out by the regional health agency through a numeric identification code. All the health data used in the study were anonymous.

### Definition of the individual deprivation index through the census data

For the definition of the individual DI, polychoric principal component analysis (PCA) was employed, and four census variables were used: citizenship, family type, employment status, and education. Table 1 reports the value attributed to each individual variable category.

**Table 1:** Census variable used to define the individual DI

Citizenship	Family Type	Employment Status	Education
1—Italian	1—Couple without children	1—Employed	1—Academic Diploma 2nd Level/Academy of Fine Arts Diploma/Master degree
2—Foreigner	2—Single-person households	2—Recipient(s) of one or more pensions due to previous employment or investment income	2—Academic 1st Level Diploma/Bachelor degree
	3—Couple with children	3—Students /Housewives/In other status	3—High school graduation
	4—Single parent	4—First-time job seekers	4—Middle school graduation
	5—Households with 2 or more families	5—Unemployed	5—Elementary school diploma
			6—Illiterates/Literate without educational qualification

### Statistical analysis

Generalized linear logistic regression models (GLM) were used to test associations between COVID-19 outcomes (tested positive, being hospitalised due to COVID-19, being admitted in ICU due to COVID-19 and death due to COVID-19) and geographical or individual DI. Age, sex and Charlson comorbidity index were included in the model as covariates. Multilevel logistic models were used to test associations between COVID-19 outcomes and PCA individual DI, geographical DI, and their interaction. Census sections were used as clusters for the model, each of which was assigned a geographical DI. Age, sex and Charlson comorbidity index were included in the model as covariates. To build the model, we followed the following steps also explained in Additional file 1: Figure S2: Preliminary step: Data preparation (centring of variables); Step1: Construction of an empty model to assess the variation of log-odds from cluster to cluster. This model did not contain the covariates. Step2: Assessment of the variation of lower-level effects from cluster to cluster. In order to perform the assessment, first, a constrained intermediate model was run, secondly, an augmented intermediate model was run and finally both were compared by performing a likelihood- ratio test. Both models contained the covariates, geographical and PCA individual DI. The augmented intermediate included also the residual term associated with the PCA individual DI, thereby estimating the random slope. Step3: Construction of a final model adding the cross-level interaction. The methodology is described in detail in the work of Morselli et al. [30]. When the variation of log-odds from cluster to cluster was not significant, the results of one-level regression analyses were considered.

The statistical analysis has been carried out separately for the first epidemic wave and the second epidemic wave.

R (version 4.2.0) was used for all statistical analysis and a p-value of 0.05 was applied for testing statistical significance.

## Results

In the study period 129,779 individuals aged more then 34 (49.1% female and 50.9% male) were tested for COVID-19: 7,160 (5.6%) during the first wave, 20,523 (15.9%) during the summer and 102,096 (78.4%) during the second wave (Additional file 1: Figure S3). In our study population the median age was 60. 24,466 (18.9%) individuals lived in a census area whose geographical DI was 1, 23,594 (18.2%) in a census area whose geographical DI was 2, 25,508 (19.6%) in a census area whose geographical DI was 3, 27,676 (21.3%) in a census area whose geographical DI was 4, 28,497 (20.5%) in a census area whose geographical DI was 5. Table 2 describes study population characteristics by epidemic wave.

**Table 2:** Description of the study population by wave of epidemics. Proportion calculated only on complete data

	1st Wave										2nd Wave									
	Negative		Positive		Hospitalization		ICU		Death		Negative		Positive		Hospitalization		ICU		Death	
	N %		N %		N %*		N %*		N %*		N %		N %		N %*		N %*		N %*	
*Sex*																				
Male	2,279 (63.3%)		1,322 (36.7%)		750 (56.7%)		165 (12.5%)		273 (20.7%)		24,075 (48.7%)		25,356 (51.3%)		3,753 (14.8%)		718 (2.8%)		1,027 (4.1%)	
Female	2,200 (63.6%)		1,259 (36.4%)		575 (45.7%)		69 (5.5%)		223 (17.7%)		25,197 (47.9%)		27,428 (52.1%)		2,542 (9.3%)		323 (1.2%)		636 (2.3%)	
NA	73 (73.0%)		27 (27.0%)		13 (48.1%)		3 (11.1%)		8 (29.6%)		28 (70.0%)		12 (30.0%)		2 (16.7%)		0 (0.0%)		1 (8.3%)	
*Age group*																				
35–69	2,316 (62.2%)		1,410 (37.8%)		536 (38.0%)		97 (6.9%)		73 (5.2%)		32,982 (45.7%)		39,241 (54.3%)		2,773 (7.1%)		480 (1.2%)		307 (0.8%)	
> 69	2,236 (65.1%)		1,198 (34.9%)		802 (66.9%)		140 (11.7%)		431 (36.0%)		16,318 (54.6%)		13,555 (45.4%)		3,524 (26.0%)		561 (4.1%)		1,357 (10.0%)	
*Comorbidity index*																				
0	3,658 (61.2%)		2,321 (38.8%)		1,144 (49.3%)		214 (9.2%)		395 (17.0%)		44,636 (47.2%)		49,996 (52.8%)		5,523 (11.0%)		927 (1.9%)		1,379 (2.8%)	
1	666 (74.8%)		224 (25.2%)		150 (67.0%)		17 (7.6%)		81 (36.2%)		3,512 (62.0%)		2,148 (38.0%)		615 (28.6%)		98 (4.6%)		224 (10.4%)	
2	190 (79.8%)		48 (20.2%)		38 (79.2%)		5 (10.4%)		26 (54.2%)		745 (68.7%)		340 (31.3%)		128 (37.6%)		13 (3.8%)		50 (14.7%)	
3	29 (90.6%)		3 (9.4%)		3 (100.0%)		0 (0.0%)		2 (66.7%)		67 (82.7%)		14 (17.3%)		4 (28.6%)		1 (7.1%)		1 (7.1%)	
NA	9 (42.9%)		12 (57.1%)		3 (25.0%)		1 (8.3%)		0 (0.0%)		340 (53.3%)		298 (46.7%)		27 (9.1%)		2 (0.7%)		10 (3.4%)	
*Geographical deprivation index*																				
1	893 (63.0%)		525 (37.0%)		267 (50.9%)		45 (8.6%)		107 (20.4%)		10,954 (58.5%)		7,761 (41.5%)		898 (11.6%)		140 (1.8%)		234 (3.0%)	
2	834 (63.4%)		481 (36.6%)		256 (53.2%)		52 (10.8%)		93 (19.3%)		9,839 (53.4%)		8,576 (46.6%)		1,005 (11.7%)		162 (1.9%)		282 (3.3%)	
3	889 (61.8%)		549 (38.2%)		269 (49.0%)		40 (7.3%)		93 (16.9%)		9,752 (48.5%)		10,357 (51.5%)		1,215 (11.7%)		185 (1.8%)		325 (3.1%)	
4	919 (65.3%)		489 (34.7%)		255 (52.1%)		51 (10.4%)		97 (19.8%)		9,472 (42.6%)		12,784 (57.4%)		1,435 (11.2%)		237 (1.9%)		355 (2.8%)	
5	1,013 (64.2%)		564 (35.8%)		291 (51.6%)		49 (8.7%)		114 (20.2%)		9,277 (41.1%)		13,302 (58.9%)		1,741 (13.1%)		316 (2.4%)		467 (3.5%)	
NA	4 (100.0%)		0 (0.0%)		0 (0.0%)		0 (0.0%)		0 (0.0%)		6 (27.3%)		16 (72.7%)		3 (18.8%)		1 (6.2%)		1 (6.2%)	
*PCA individual deprivation index*																				
1	812 (59.8%)		546 (40.2%)		232 (42.5%)		47 (8.6%)		47 (8.6%)		13,129 (51.0%)		12,611 (49.0%)		923 (7.3%)		155 (1.2%)		113 (0.9%)	
2	527 (59.8%)		355 (40.2%)		150 (42.3%)		25 (7.0%)		23 (6.5%)		7,764 (44.0%)		9,884 (56.0%)		713 (7.2%)		129 (1.3%)		86 (0.9%)	
3	938 (65.7%)		490 (34.3%)		245 (50.0%)		58 (11.8%)		85 (17.3%)		10,045 (50.0%)		10,028 (50.0%)		1,345 (13.4%)		266 (2.7%)		385 (3.8%)	
4	1,098 (63.8%)		622 (36.2%)		358 (57.6%)		59 (9.5%)		187 (30.1%)		9,922 (48.6%)		10,481 (51.4%)		1,604 (15.3%)		261 (2.5%)		532 (5.1%)	
5	1,177 (66.4%)		595 (33.6%)		353 (59.3%)		48 (8.1%)		162 (27.2%)		8,440 (46.3%)		9,792 (53.7%)		1,709 (17.5%)		229 (2.3%)		547 (5.6%)	

^*^ The percentage of hospitalization, ICU and deaths are referred to people positive to the COVID-19

Comorbidity index: 0: no comorbidity, 4: highest level of comorbidity. Index of geographical and PCA individual deprivation: 1: lowest level of deprivation, 5: highest level of deprivation

### PCA Individual deprivation index

The census variables selected to build the individual DI were available for a subpopulation of 129,779 individuals. We have retained two principal components in our PCA: the variance explained by the principal component 1 was 34.1% and the variance explained by the principal component 2 was 25.5% (Additional file 1: Figure S4). The coefficients forming these components can be found in Additional file 1: Table S1.

### Outcomes of interest by type of deprivation index

**Fig. 2:**
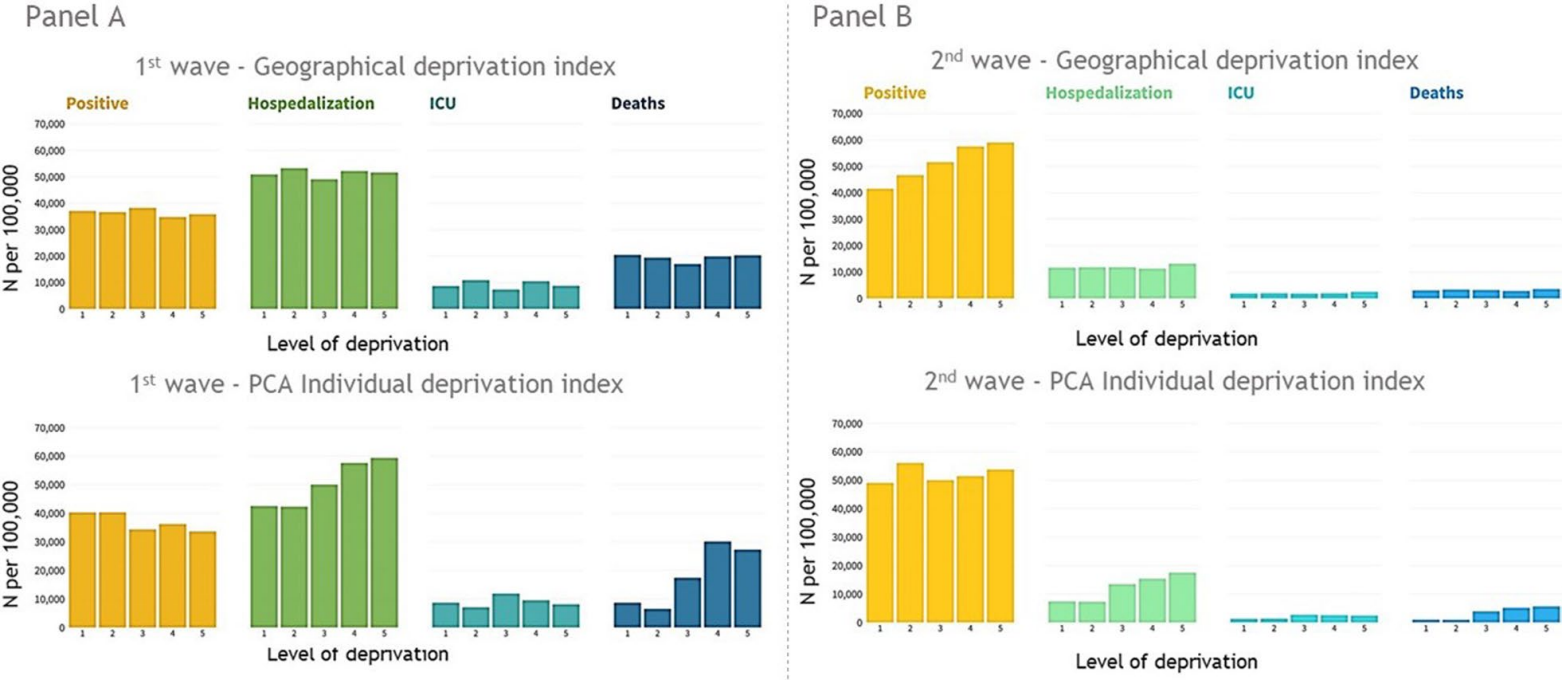
Counts per 100,000 of positive tests, hospitalizations, ICU admissions, and deaths per DI level. A: 1st wave (20 February 2020 to 31 May 2020). B: 2^nd^ wave (16 September 2020 to 31 December 2020)

Figure 2 shows the counts of positive tests, COVID-19 hospitalisations, intensive care unit admissions and deaths among the DI groups per 100,000 tests or positive tests during the first or second wave respectively.

#### 1st epidemic wave

**Fig. 3:**
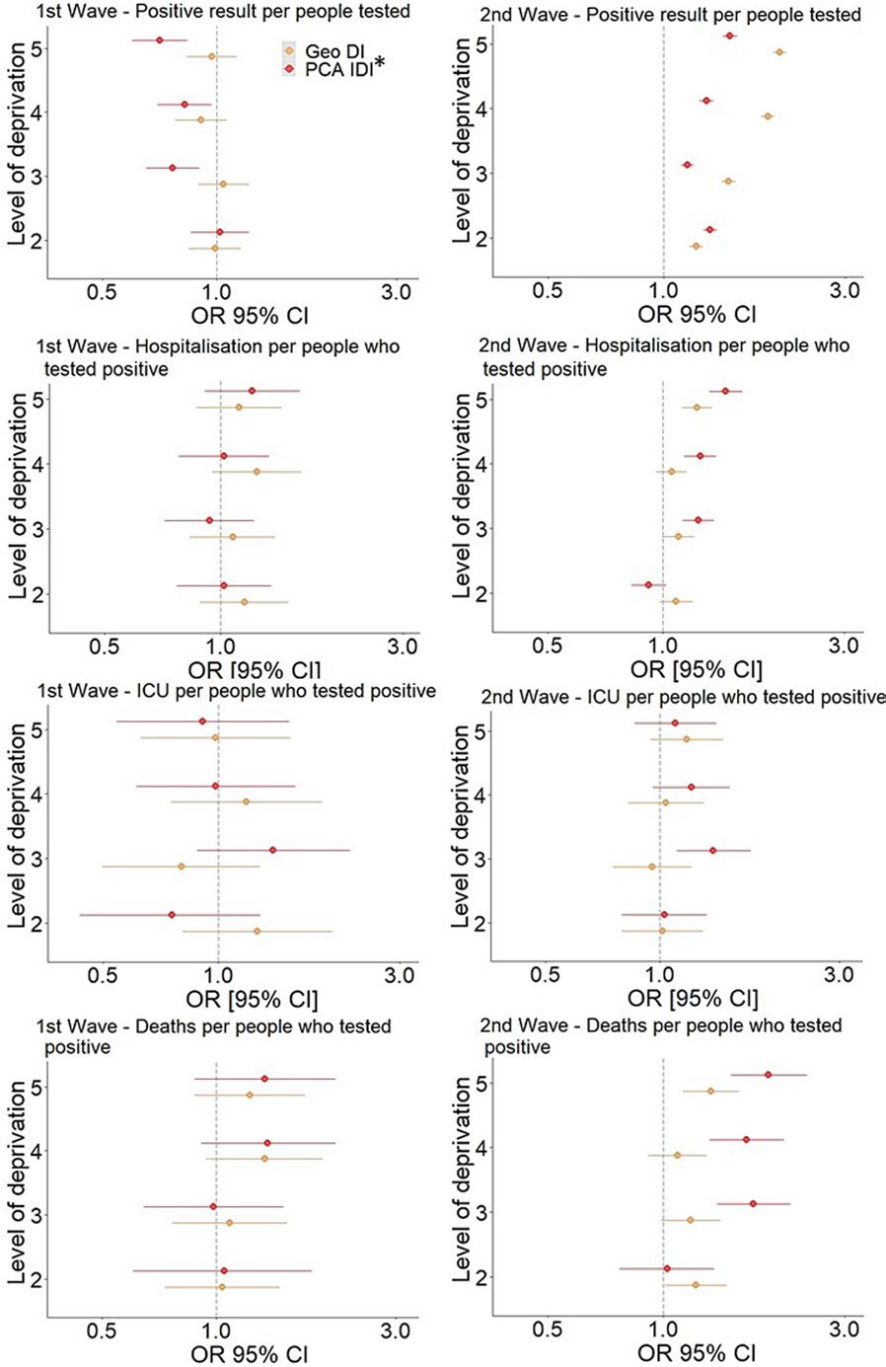
Adjusted ORs per increase in the geographical, PCA individual DI for the counts of positive tests, hospitalisations, ICU admissions, and deaths. *IDI: individual deprivation index

According to the results of the logistic GLMs, during the first wave, the risk of testing positive for Sars-CoV-2 infection was not significantly different in people with a level of geographical deprivation higher than 1 when compared with the less deprived population group. The models that used PCA individual ID reported that in the more deprived population, the odds of testing positive were lower compared those estimated in the less deprived population, with ORs lower than one. Considering both DI indicators, the ORs of being hospitalised, admitted to the ICU or dying when positive did not exhibit significant differences between the subgroups of the population with the highest DI and those with the lowest DI. (Fig. 3, Table 3).

**Table 3:** Associations between geographical and individual DI and risk of Sars-CoV-2 infection and disease severity

1st Wave									2nd Wave								
	Positive per tested		Hospitalisations per positive tests		ICU per positive tests		Deaths per positive tests			Positive per tested		Hospitalisations per positive tests		ICU per positive tests		Deaths per positive tests	
Geo DI	OR	CI 95%	OR	CI 95%	OR	CI 95%	OR	CI 95%	Geo DI	OR	CI 95%	OR	CI 95%	OR	CI 95%	OR	CI 95%
1	Ref.		Ref.		Ref.		Ref.		1	Ref.		Ref.		Ref.		Ref.	
2	0.99	(0.84–1.16)	1.16	(0.89–1.51)	1.27	(0.81–1.99)	1.04	(0.73–1.48)	2	1.22	(1.17–1.27)	1.08	(0.98–1.20)	1.02	(0.79–1.30)	1.22	(1.01–1.47)
3	1.04	(0.90–1.21)	1.08	(0.83- 1.39)	0.80	(0.50–1.28)	1.09	(0.77–1.54)	3	1.48	(1.42–1.54)	1.10	(0.99–1.21)	0.96	(0.75–1.21)	1.18	(0.99–1.42)
4	0.91	(0.78–1.06)	1.24	(0.95–1.63)	1.19	(0.75.-1.87)	1.34	(0.94–1.92)	4	1.88	(1.80–1.95)	1.06	(0.96–1.16)	1.04	(0.83–1.31)	1.09	(0.91–1.30)
5	0.97	(0.84–1.13)	1.12	(0.87–1.45)	0.98	(0.62–1.55)	1.23	(0.88–1.73)	5	2.02	(1.94–2.10)	1.23	(1.12–1.35)	1.18	(0.95–1.47)	1.34	(1.05–1.58)

PCA Individual DI									PCA Individual DI								
1	ref		ref		ref		ref		1	ref		ref		ref		ref	
2	1.02	(0.86–1.22)	1.02	(0.77–1.36)	0.76	(0.44–1.30)	1.05	(0.61–1.81)	2	1.33	(1.28–1.38)	0.92	(0.83–1.36)	1.03	(0.79–1.33)	1.02	(0.77–1.36)
3	0.77	(0.65–0.90)	0.94	(0.72–1.23)	1.40	(0.88–2.21)	0.98	(0.64- 1.51)	3	1.16	(1.11–1.20)	1.24	(1.13–1.38)	1.39	(1.11–1.74)	1.73	(1.38–2.16)
4	0.83	(0.70–0.97)	1.02	(0.78- 1.34)	0.98	(0.61–1.59)	1.37	(0.91–2.06)	4	1.30	(1.25–1.35)	1.26	(1.14–1.62)	1.21	(0.96–1.54)	1.65	(1.32–2.07)
5	0.71	(0.60–0.84)	1.21	(0.91–1.61)	0.91	(0.54–1.53)	1.34	(0.88–2.06)	5	1.49	(1.43–1.56)	1.46	(1.32–1.84)	1.10	(0.86–1.41)	1.89	(1.50–2.37)

#### 2nd epidemic wave

According to the results of the logistic GLMs, during the 2nd epidemic wave the risk of testing positive for Sars-CoV-2 infection was significantly higher in people with a level of deprivation higher than 1 when compared with the less deprived population group, with generally homogeneous results disregarding the DI used in the model. For what concern the odds of being hospitalised and dying if positive, considering the geographical DI, the subgroup with deprivation level 5 has a higher OR of being hospitalised (OR: 1.23; 95% CI 1.12–1.35) and dying (OR: 1.34; 95% CI 1.05–1.58) if positive than the subgroup with deprivation level 1. Calculating GLMs with the PCA individual DI, the OR of being hospitalised and dying if positive increased with increasing individual deprivation level also in population subgroups with a DI less than 5. The models that used individual ID reported that as deprivation increased, the risk of hospitalisation and death increased, which was not evident in the models that used geographic ID. The OR of being admitted to the ICU when positive in the subgroups of the population with the highest DI was not significantly different from the subgroup of the population with the lowest DI, irrespective of the type of DI used (Fig. 3, Table 3).

### Multilevel logistic modelling

#### 1st epidemic wave

The proportion of the between-cluster variation (ICC) in the probability of being positive if tested during the first wave was 9.9 (Table 4). This indicates that 9.9% of the chances was explained by between the census section differences. The deviance of the augmented intermediated model was significantly lower than the deviance of the constrained model. Table 4 reports multilevel logistic regression results. The model showed that higher level PCA individual DI were significantly associated with lower probability of being positive if tested. Additional file 1: Figure S5, Panel A shows the interaction between geographical ad individual DI in the prospective prediction of being positive if tested. Although the association between geographical and individual DI was not significant, the coefficient (ß) was negative. The proportion of the between-cluster variation in the probability of being hospitalised, admitted to ICU and dying if positive during the first wave was respectively 5.4, 4.9 and 2.1. Therefore, the results for the one-level regression model were considered.

**Table 4:** Results of multilevel logistic regression or GLM models including geographical and individual DI interaction

	1st Wave		2nd Wave	
	Outcome: being positive if tested			
ICC for empty models	0.10		0.15	
	ß	SE	ß	SE
PCA Individual DI	− 0.12***	0.03	0.02°	0.01
Geo DI	− 0.01	0.02	0.04***	0.01
Female	− 0.02	0.05	− 0.01	0.01
Age	0.01**	0.00	− 0.01***	0.00
Charlson comorbidity index 1	− 0.66***	0.09	− 0.61***	0.03
Charlson comorbidity index 2	− 0.96***	0.17	− 0.89***	0.07
Charlson comorbidity index 3	− 1.69**	0.62	− 1.77***	0.30
Geo x Individual DI	0.00	0.01	0.00	0.00

^***^ p-value < 0.001; ** p-value = 0.001; * p-value = 0.01; ° p-value = 0.05

#### 2nd epidemic wave

The proportion of the between-cluster variation in the probability of being positive if tested during the first wave was 15.2. This indicates that 15.2% of the chances was explained by between the census section differences. The deviance of the augmented intermediated model was significantly lower than the deviance of the constrained model. The model shows that both higher level PCA individual DI or geographical DI were significantly associated with higher probability of being positive if tested (Table 4). Additional file 1: Figure S5, Panel B shows the interaction between geographical ad individual DI in the prospective prediction of being positive if tested. Although the association between geographical and individual DI was not significant, the coefficient (ß) was positive. The proportion of the between-cluster variation in the probability of being hospitalised, admitted to ICU and dying if positive during the first wave was respectively 1.3, 2.4 and 1.9. Therefore, the results for the one-level regression model were considered.

## Discussion

This study reports results from a large population-wide cohort of people tested for COVID-19 in the Apulia region, Italy, during the first and second wave of the pandemic in 2020. To our knowledge, this was the first study to investigate the role of individual DI on COVID-19 outcomes in Italy. Previous studies were limited to the use of an area-based DI or the individual component of socioeconomic deprivation as a proxy of individual deprivation [17, 31–33]. Although for the first wave, the geographic DI and the individual DI calculated in this study show no differences in the non-significance of the association with COVID-19 outcomes, the results were different for the second wave.

According to our findings, the association between the risk of testing positive for Sars-CoV-2 and the level of socioeconomic deprivation in the Apulia region changed between the first and second waves. According the analysis done with the PCA individual DI, during the first wave, there was a significant inversely proportional trend between the DI and the risk of testing positive. As deprivation increased, the risk of being positive when tested decreased. During the second wave, individuals with a higher level of socio-economic deprivation had a higher statistically significant probability of testing positive. This may be explained by the fact that during the early stages of the epidemic outbreak, with affected geographical areas still circumscribed, most of the cases in the Apulia region were due to returning residents [34]. Indeed, the gradual implementation of control measures in Italy sparked substantial movements of people travelling from northern regions at the epicentre of the epidemic toward other regions, such as Apulia. Then, the swift extension of lockdown to the entire country [22] mitigated the impact of these COVID-19 seeding events and the epidemic in Apulia was successfully contained. For these reasons, during the first wave in Apulia, the epidemic probably spread mainly among individuals who had the financial means to travel or who were economic migrants in Lombardy or northern regions, areas of intense economic activities. During the first wave, individuals with a higher level of socio-economic deprivation did not have a statistically significant higher probability of being hospitalised or dying if positive. These results were in line with those reported by Furtunato et al. [35]. While during the second wave, individuals with a higher level of socio-economic deprivation had a higher statistically significant higher probability of being hospitalised or dying if positive.

Concerning the second wave, the two DI showed a clear association between the probability of testing positive and the level of deprivation. However, the geographic DI showed a stronger trend. The results of this study showed that most people with a higher individual DI live in the most deprived census areas. Since people with a higher individual DI were more often in jobs that were less amenable to remote working and so they benefited less from lockdown restrictions than those able to work from home; they had a higher likelihood of acquiring the infection [36]. Moreover, they generally live in higher-density environments where family members could be infected secondarily [37]. This may have meant that in more deprived areas the virus circulated more widely than in less deprived areas. As individual DI increased, so did the probability of hospitalisation and death. The same trend was not observed for geographical DI, which showed a significantly increased probability of being hospitalised or dying if positive only for the highest level of deprivation. In accordance with our results, Mateo-Urdiales and colleagues, using the same geographical DI used in this study, but at the municipality level, found a higher incidence of cases in the most deprived municipalities compared with the least deprived ones and no differences in case-hospitalisation and case-fatality according to deprivation were observed in any period under study. The same result has also been reported by other studies using geographical measures of deprivation conducted in Spain [38, 39]. While many factors could explain this finding, an alternative could be that hospitalisation and death cases were similar across areas with different levels of deprivation in a well-developed universal healthcare system, such as Italy and Spain.

The use of individual DI enabled to understand that actually the most disadvantaged people had a higher risk of hospitalisation and death, regardless of the area in which they lived. To avoid exacerbating existing social inequalities and marginalisation, it is essential to be able to monitor them. According to our results, using geographical DI as a proxy for individual DI may lead to inaccurate assessments. However, the geographic DI can have a relevance in providing insight into the distribution of infection within different neighbourhoods, accounting for their heterogeneity. This may have important implications for public health action planning.

Understanding the relationship between deprivation and COVID-19 outcomes is multifaceted and complex. This is why the secondary objective of this manuscript was to test the hypotheses about how individual and geographical DI interact to predict COVID-19 outcomes. According to the results of the multilevel logistic and GLM models, there was no association between COVID-19 outcomes and the interaction between PCA individual DI and geographical DI. Although this may seem at odds with the robust evidence in the literature demonstrating an interaction between socioeconomic status in one's neighbourhood, individual deprivation and health, this evidence is mainly based on health outcomes of non-communicable chronic diseases [40–43].

The COVID-19 pandemic has been described as a syndemic pandemic [44]. Originating in anthropology, a syndemic describes a set of closely intertwined and mutual enhancing health problems that significantly affect the overall health status of a population within the context of a perpetuating configuration of noxious social conditions [45]. Deprivation—which is an area measure of poverty, low income, and a reflection of the wider social determinants of health (such as housing, working conditions, unemployment, health-care access, etc.)— results in multiple, interacting, and additive adverse risk factors for COVID-19 outcomes. These can be summarised by way of four inter-related pathways: unequal exposure, unequal transmission, unequal vulnerability, and unequal susceptibility [46]. Living in a more deprived neighbourhood may increase the likelihood of exposure and transmission. Although it has been proven that individuals living in more deprived neighbourhoods have worse profiles on many components of subjective health, more risk factors and higher morbidity and mortality rates than their counterparts living in less deprived neighbourhoods, the level of neighbourhood deprivation does not influence with a higher extent the health of those with low individual DI compared to those whit a higher individual DI [43]. This could be the reason why not all individuals living in the most deprived neighbourhoods have a higher vulnerability and susceptibility to COVID-19 and would explain why the analyses in this study give different results when using geographical DI than when using individual DI.

This study had some limitations. Firstly, policies for the execution of the diagnostic test changed during the period under study. During the first wave, the diagnostic capacity was limited, and the number of positives could be under-reported, whereas for the second wave data could represent laboratory-confirmed COVID-19 cases who sought care. Secondly, the death status only reflects deaths occurring in individuals diagnosed with COVID-19, but this number may be underestimated due to non-diagnosis. Third, the Charlson comorbidities index was utilized as a comorbidity index, though it may not be the most suitable or up-to-date choice for studies related to SARS-CoV-2. Unfortunately, we could not independently calculate a comorbidity index due to a lack of available data. Finally, the DI is a composite measure of deprivation, and it is difficult to know which of its constituent factors are driving associations. There are other potential variables, such as income and homeownership, that could enhance the deprivation index, but we were unable to assess them due to information unavailability.

## Conclusion

Historically, pandemics have been experienced unequally with higher rates of infection and mortality among the most disadvantaged communities [47]. Evidence from a variety of countries suggests that these inequalities are being mirrored in the COVID-19 pandemic [36, 48] and this study also adds to this evidence. The causal forces at work at the interface of disease epidemics and social inequality are complex: they are operant at the level of individuals, neighbourhoods, and local communities. The results of this study remind us to be cautious about using geographical DI as a proxy for the level of individual social disadvantage because of the inevitable potential ecological bias that can result from attributing a collective measure to an individual and may lead to inaccurate assessments.

The geographical DI is often used due to a lack of individual data. However, on the determinants of health and health inequalities, monitoring has to have a central focus. Health inequalities monitoring provides evidence on who is being left behind and informs equity-oriented policies, programmes and practices [49]. The emergence of novel data sources and monitoring approaches for public health surveillance is triggered by technological advances in data retrieval and data analysis. Unfortunately, the information richness resulting from the data science revolution is not exploited to better understand health inequities and inform the development and implementation of services and policies that tackle inequities in health [50, 51].

Reducing these inequalities—and those that may result from future pandemics—requires long-term action to improve equity in health and wealth. Future research and data collection should focus on improving surveillance systems, for example by integrating individual measures of inequalities into national health information systems. Understanding the social nature of emerging infectious disease pandemics will ultimately help reduce the burden of disease.

### Supplementary Information

**Additional file 1: Figure S1**. The geographical boundaries of Italy and its regions with the Apulia region in red. **Figure S2.** Summary of the three steps performed to build the multilevel logistic regression. **Figure S3.** Epidemic curve of positive COVID-19 PCR test results (7 days moving average). **Figure S4.** Scree plot of PCA performed on census variables. **Figure S5.** Interaction between geographical ad individual DI in the prospective prediction of being positive if tested. Estimated values reflect statistical adjustment for sex, age, and Charlson comorbidity index. **Table S1.** Coefficients forming components retained in the PCA.

## Data Availability

The data that support the findings of this study are available from Strategic Regional Health and Social Agency of Puglia (AReSS Puglia) but restrictions apply to the availability of these data, which were used under license for the current study, and so are not publicly available. Data are however available from the authors upon reasonable request and with permission of AReSS Puglia.

## Abbreviations

DI: Deprivation Index
PCA: Principal component analysis
SEP: Socioeconomic position
SDH: Social determinant of health
SSN: Servizio Sanitario Nazionale
R and AP: Regions and two autonomous provinces
ICU: Intensive care unit
GLM: Generalized linear logistic regression models
OR: Odds Ratio
CI: Confidence Interval
