# Supplementary material for: Comparison of geographical and individual deprivation index to assess the risk of Sars-CoV-2 infection and disease severity: a retrospective cohort study
Source: Int J Health Geogr. 2024 Apr 4;23:8. doi: 10.1186/s12942-024-00367-6 (PMC10993505; doi:10.1186/s12942-024-00367-6)

Supp. Material Figure 1. The geographical boundaries of Italy and its regions with the Apulia region in red.


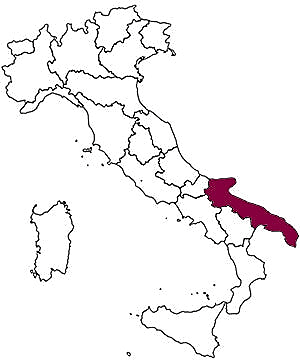


Supp. Material Figure 2. Summary of the three steps performed to build the multilevel logistic regression.


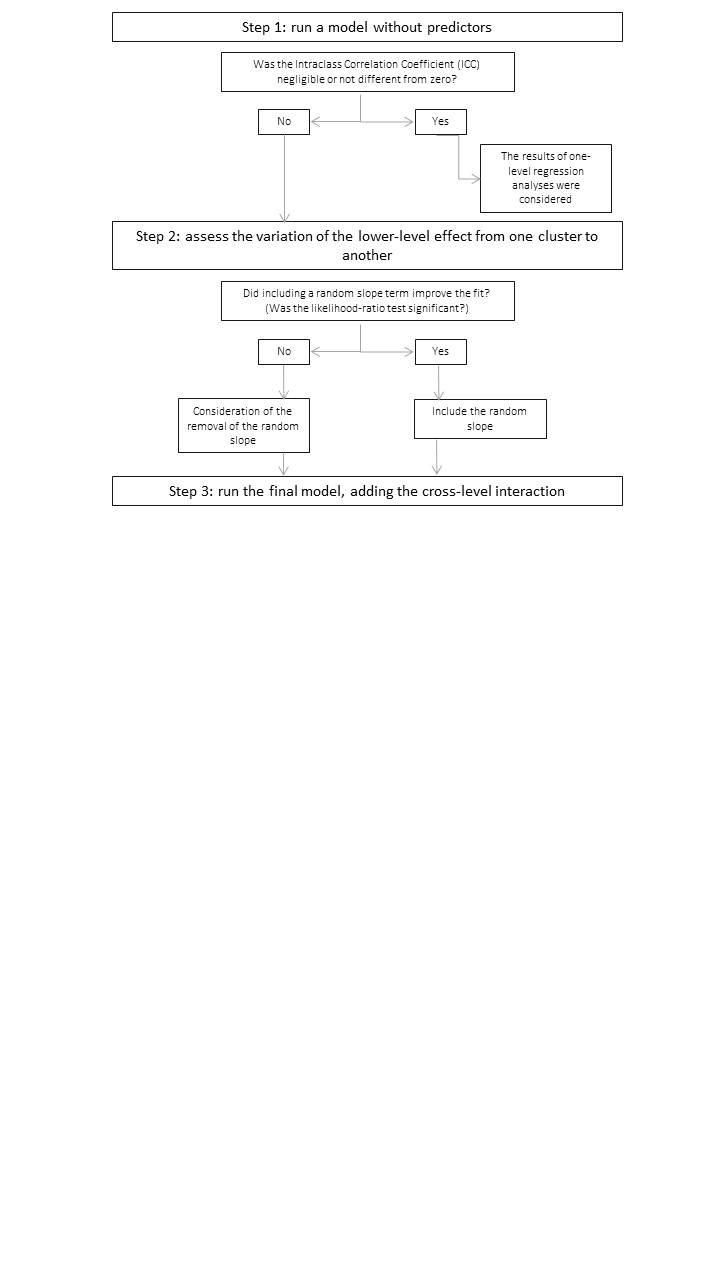


Supp. Material Figure 3. Epidemic curve of positive COVID-19 PCR test results (7 days moving average).


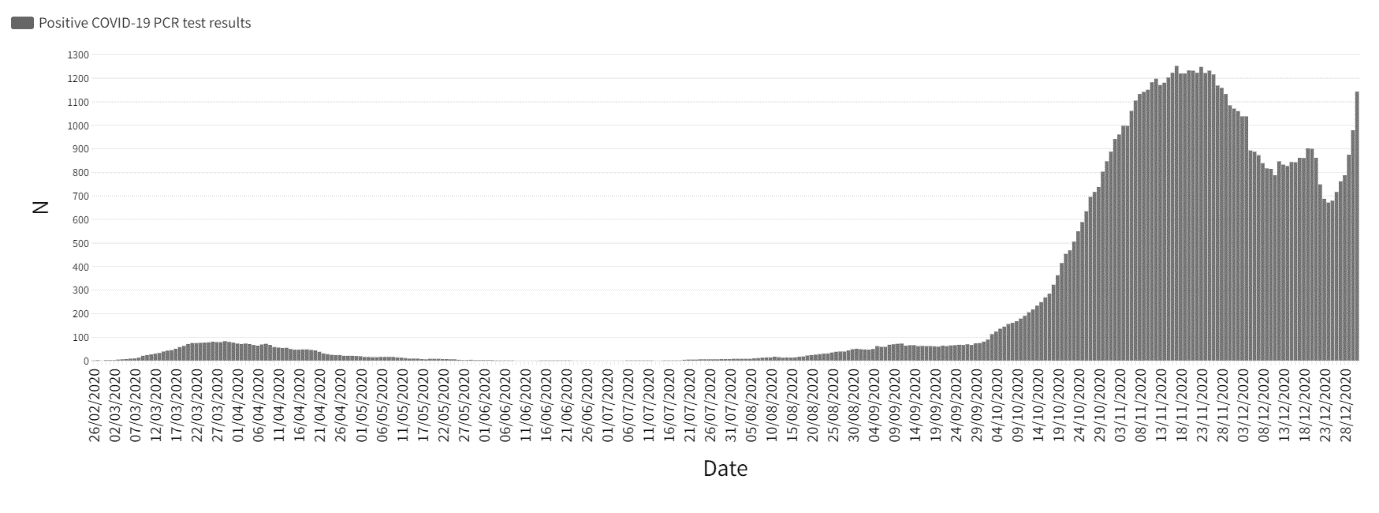


Supp. Material Figure 4. Scree plot of PCA performed on census variables.


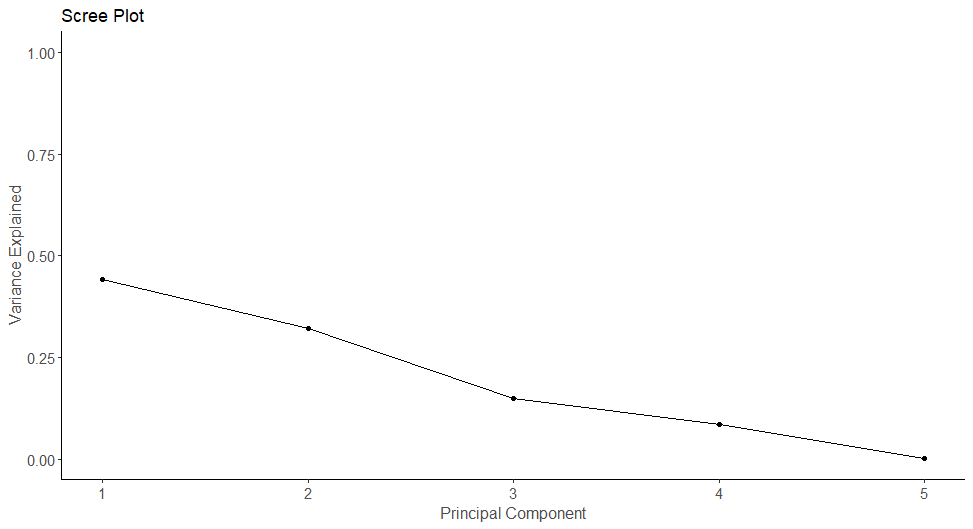


Supp. Material Table 1. Coefficients forming components retained in the PCA.

|  | PC1 | PC2 |
| --- | --- | --- |
| Citizenship | 0.08 | 0.62 |
| Family Type | -0.16 | 0.79 |
| Employment Status | 0.81 | 0.15 |
| Education | 0.79 | -0.21 |

Supp. Material Figure 5. Interaction between geographical ad individual DI in the prospective prediction of being positive if tested. Estimated values reflect statistical adjustment for sex, age, and Charlson comorbidity index.


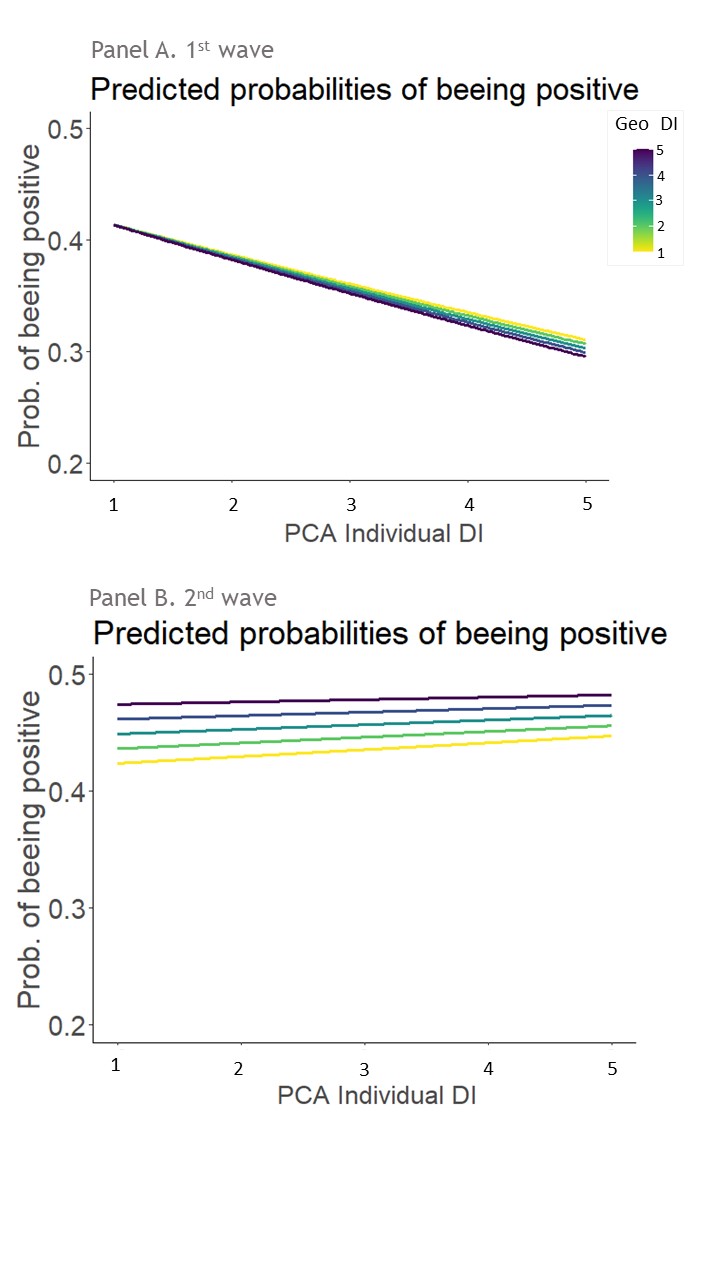

Supplement: Supplementary file 1 — Additional file 1: Figure S1. The geographical boundaries of Italy and its regions with the Apulia region in red. Figure S2. Summary of the three steps performed to build the multilevel logistic regression. Figure S3. Epidemic curve of positive COVID-19 PCR test results (7 days moving average). Figure S4. Scree plot of PCA performed on census variables. Figure S5. Interaction between geographical ad individual DI in the prospective prediction of being positive if tested. Estimated values reflect statistical adjustment for sex, age, and Charlson comorbidity index. Table S1. Coefficients forming components retained in the PCA. [file 12942_2024_367_MOESM1_ESM.docx]
